# Analysis of selected digital risks as a basis for developing digital hygiene strategies. A perspective of vulnerable populations

**DOI:** 10.3389/fpubh.2026.1932831

**Published:** 2026-09-23

**Authors:** Milena Wojciechowska, Magdalena Mateja, Agata Żaroń, Marija Spevan, Silvije Šegulja

**Affiliations:** 1 Department of Law and Health Policy, Ludwik Rydygier Collegium Medicum in Bydgoszcz, University of Nicolaus Copernicus in Toruń, Toruń, Poland; 2 Department of Communication, Media and Journalism, Institute of Information and Communication Research, Nicolaus Copernicus University in Toruń, Toruń, Poland; 3 Department of Health Economics, Ludwik Rydygier Collegium Medicum in Bydgoszcz, University of Nicolaus Copernicus in Toruń, Toruń, Poland; 4 Faculty of Health Studies, University of Rijeka, Rijeka, Croatia

**Keywords:** cybersecurity, digital hygiene promotion, digital risks, prevention, problematic internet use

## Abstract

The rapid development of digital technologies and widespread Internet access have transformed modern societies, creating new opportunities alongside emerging public health challenges. Digital risks, including problematic Internet and social media use, gaming disorder and problematic gaming, cyberbullying, online sexual risks, online gambling, and cybersecurity threats, may adversely affect psychological wellbeing, social functioning, and quality of life, particularly among vulnerable populations. This narrative review examined selected digital risks and their implications for promoting digital hygiene within public health prevention frameworks. A total of 64 publications published between 2012 and 2026 were included. Relevant literature was identified through PubMed, Scopus, and Web of Science, supplemented by reference-list searching and selected reports from international and European institutions. Literature was selected purposively, with priority given to systematic reviews, meta-analyses, epidemiological studies, and relevant conceptual and theoretical publications. The evidence was synthesized narratively and thematically, focusing on epidemiology, vulnerable populations, digital risks, risk and protective factors, and public health prevention and policy. The review focused on children and adolescents, older adults, individuals with mental health conditions, and socioeconomically disadvantaged populations. The findings indicate that digital risks arise from complex interactions among individual, interpersonal, digital-environmental, and structural factors. Effective prevention therefore requires a multidimensional, socio-ecological approach integrating digital and health literacy, self-regulation, family- and school-based prevention, community support, safer digital environments, and appropriate policy measures. Digital hygiene should be considered an emerging component of public health practice, with further research needed to develop and evaluate population-specific, multilevel interventions.

## Introduction

The rapid advancement of digital technologies and the widespread accessibility of the Internet have fundamentally transformed contemporary society, creating unprecedented opportunities for communication, education, employment, and access to information. At the same time, the increasing integration of digital tools into everyday life has given rise to a broad range of digital risks that may adversely affect mental health, wellbeing, and social functioning. Despite its undeniable benefits, the digital environment has also emerged as a source of complex public health challenges, including excessive and problematic technology use, impaired control over online behaviors, cyberbullying, information overload, misinformation, and threats to users' privacy and digital security (1).

From a public health perspective, one of the most significant consequences of excessive or maladaptive engagement with digital technologies is the growing prevalence of behavioral (non-substance) addictions related to digital activities. Problematic use of the Internet, social media platforms, digital games, and other forms of online engagement has become an important public health challenge, underscoring the need for comprehensive prevention strategies and evidence-based educational interventions that promote healthy and balanced digital behaviors. These challenges may adversely affect not only individuals' mental health but also their social relationships, educational attainment, occupational functioning, and overall quality of life (2, 3).

Current research suggests that the relationship between different factors is more complicated and requires more of a network analysis of various factors rather than simple and detailed analysis of individual relationships (4, 5). Due to the numerous factors that moderate the relationship between specific risk factors, protective factors, and outcomes, we decided to show a broad perspective that aligns with the Bronfenbrenner's Ecological Systems Model (6, 7). According to that model, the considered risk factors and protective factors should be analyzed at different layers—from the individual level, through interpersonal relationships to the broader culture you live in.

Various terms have been proposed to describe addictions associated with digital technologies, including: netaholism, Internet addiction, cyberaddiction, Internet dependency, computer addiction, disorders caused by Internet addiction, and infoholism (2, 8). The wide range of terminology used to describe these conditions reflects their multifaceted nature. However, the lack of a standardized nomenclature limits the comparability of research findings, hampers the accumulation of robust scientific evidence, and remains a significant methodological challenge in this field (9). Despite ongoing definitional inconsistencies, these conditions are generally classified as behavioral addictions, characterized by repetitive and difficult-to-control digital behaviors that may result in adverse health and social consequences (8). Clinical studies indicate similarities in the underlying neurobiological mechanisms of these behaviors and those observed in substance use disorders, particularly with regard to the reward system, impulse control, and behavioral reinforcement processes (2). Kuss et al. (10) conducted a systematic review of 18 neuroimaging studies, demonstrating clear similarities across different types of addiction, particularly between substance use disorders and Internet and gaming addiction, and across multiple levels of analysis. At the molecular level, Internet addiction is characterized by a general reward deficiency associated with reduced dopaminergic activity.

Among specific forms of digital addiction, one can distinguish Internet social addiction (cyber-relational addiction), characterized by excessive involvement in online relationships and interactions at the expense of face-to-face contact, which may lead to weakened real-world social bonds (2). Problematic engagement with digital technologies encompasses a range of behaviors, including excessive internet use, problematic social media use, gaming disorder, and online gambling. These activities share common addictive features, such as impaired control, compulsive engagement, and continuation despite negative consequences (9).

Effective prevention of digital risks requires the early identification of risk factors and a comprehensive understanding of the mechanisms underlying their development. Such knowledge provides the foundation for designing evidence-based preventive interventions. From a public health perspective, digital hygiene—understood as a set of practices that promote safe, responsible, and health-promoting use of digital technologies—has emerged as an important component of health promotion and digital risk prevention (11). The promotion of digital hygiene can be regarded as an integral component of contemporary public health strategies aimed at enhancing digital competencies, fostering informed user behaviors, and reducing the adverse health and social consequences associated with inappropriate technology use (12). The development of effective digital hygiene promotion strategies requires a shift away from approaches focused solely on mitigating the consequences of maladaptive technology use toward interventions grounded in the systematic identification and analysis of emerging risks. A structured assessment of digital risks enables the design of more targeted educational and preventive programs aimed at strengthening digital literacy, promoting responsible online behavior, and reducing negative health and social outcomes (10, 11).

We strongly encourage to take into account all layers of functioning when organizing public health interventions. We believe that it is very important to utilize all opportunities for mental health protection in the area of cyber risk and we would like to encourage policymakers and decision-makers to create a comprehensive intervention model, so that preventive actions are as effective as possible. However, a list of risk factors (13) was indicated, we wanted to gain a deeper understanding of this complex problem, which seems to be so important to take appropriate actions. We see the need to have a positive impact on mental health, taking into account the diversity of society, which also requires the diversification of this impact. Although digital health is an important and current topic, there is not much information on risk factors in the field of public health. In this field, the discussion rather focuses on the possibilities of supporting health or individual health components, instead of entire healthcare systems (14).

The aim of this study is to analyze selected digital risks and to determine their relevance for the development of digital hygiene promotion strategies as part of preventive interventions in public health. At the same time, it should be noted that the concept of digital hygiene and its promotion lies at the intersection of several scientific disciplines, including communication and media studies, and has attracted particular attention in the field of media education and digital competencies (1). Recognizing the advantages of an interdisciplinary approach over a narrow single-discipline perspective, this article is grounded in public health as the primary analytical framework.

## Materials and methods

### Scope and selection of literature

This narrative review examined selected digital risks and their implications for digital hygiene promotion within a public health prevention framework. Given the review's broad, multi-dimensional scope, literature was selected purposively rather than through a predefined systematic review protocol, guided by four principles: (1) methodological strength, favoring systematic reviews, meta-analyses, and large-scale epidemiological studies over single small-sample investigations; (2) recency, reflecting the fast-evolving nature of digital technologies, with a small number of foundational theoretical works (e.g., Bronfenbrenner's ecological systems theory) retained regardless of publication date for their conceptual value; (3) geographic and cultural breadth, favoring multi-country meta-analyses (spanning, in some cases, 17–31 countries) and data from international bodies (WHO, OECD, Eurostat, ENISA) alongside country-specific studies from diverse settings—including several European countries, with particular attention to Poland, as well as North America and Asia—to avoid an unduly narrow, single-region perspective; and (4) topical relevance to five predefined digital risk domains (problematic internet and social media use, gaming disorder, cyberbullying and online sexual risks, online gambling, and cybersecurity threats) and four vulnerable population groups (children and adolescents, older adults, individuals with mental health conditions, and socioeconomically disadvantaged populations).

A total of 64 publications published between 2012 and 2026 were included in the analysis. This timeframe covers a period of rapid development and widespread adoption of digital technologies, particularly smartphones, social media, and Internet-based services. The beginning of this period corresponds to a phase of rapidly increasing access to mobile devices and the Internet, while the subsequent years encompass the emergence of new patterns of technology use, problematic use behaviors, and the growing recognition of digital risks as a public health concern. Extending the timeframe to 2026 also allows for consideration of the most recent developments in the digital environment, including rapid changes in social media, mobile technologies, and artificial intelligence. This timeframe was considered appropriate for providing a current and comprehensive overview of digital risks, risk and protective factors, and opportunities for promoting digital hygiene.

Relevant peer-reviewed literature was identified through PubMed, Scopus, and Web of Science, supplemented by reference-list screening and selected reports from international and European institutions.

The evidence was organized thematically around epidemiology and vulnerable populations, major digital risks, risk and protective factors, and public health prevention and policy.

Synthesis followed a public health and socio-ecological perspective and was conducted narratively, emphasizing population-specific and cross-cutting risk and protective factors and their implications for multilevel prevention. No formal quantitative synthesis or standardized methodological appraisal was performed.

### Epidemiology of digital risks and vulnerable populations

#### Children and adolescents

Although digital health issues are usually based on the same mechanism related to the reward system, the dopamine loop, and receptor downregulation, risk factors have different significance at different ages. Children and adolescents are a particularly vulnerable group with regard to the adverse effects of intensive digital technology use. This vulnerability reflects both the intentional design of many digital platforms to maximize user engagement—particularly among younger users—and ongoing neurodevelopmental processes during this life stage. Brain maturation, including the development of neural connectivity and systems involved in self-control, emotional regulation, and decision-making, is not yet fully complete. Research conducted on preschool-aged children shows a link between increased use of screen media and the structure of the brain's surface (15) and lower microstructural integrity of white matter (16). During the period of early and rapid brain development, white matter supports language abilities and early reading skills, so the consequences may affect overall development. Social cognitive abilities also develop very early, but their significance seems particularly evident during adolescence (17). During adolescence, a temporary imbalance arises between the limbic system, which is responsible for emotions, and the prefrontal cortex, which is responsible for self-control (18). This is a time in which the fear of separation is particularly significant, and phones are used for this purpose while at the same time social skills are not being developed. The latest WHO data on problematic social media use among adolescents (11–15 years old) show a rising trend–11% exhibit signs of problematic behavior on social media, have difficulty controlling its use, and experience negative consequences. It is indicated that healthier online habits are needed (19).

As a result, young users may be more susceptible to reinforcement mechanisms embedded in digital environments, such as reward systems, notifications, and other engagement-driven design features (2, 12). Consequently, intensive exposure to digital technologies may increase the risk of problematic use patterns and associated adverse health and social outcomes.

The extent of the problem is supported by empirical evidence. In a study of 584 adolescents aged 14–18 years, problematic internet use, gaming, and social media use were assessed using standardized instruments, including the Young Internet Addiction Test, the Internet Gaming Disorder Scale, and the Bergen Facebook Addiction Scale. The results showed that 65.6% of participants exhibited symptoms of Internet addiction, 61.3% demonstrated problematic gaming behavior, and 92.8% reported addictive Facebook use. Co-occurring mental health difficulties were also observed, including depressive and anxiety symptoms, as well as social functioning problems among adolescents with problematic digital use patterns (20).

Recent studies on Polish children as internet users have similarly identified problematic patterns of social media use and gaming, including loss of control, compulsive checking, and difficulty limiting online time despite negative consequences. According to OECD estimates, around 10% of adolescents in OECD countries report problematic social media use, and evidence synthesized by the OECD indicates that problematic internet use is associated with sleep disturbances, attention and cognitive difficulties, and poorer mental health outcomes, including symptoms of depression and anxiety (21). Other studies further confirm variability in prevalence estimates depending on population characteristics, age, and diagnostic criteria. In a sample of 638 adolescents, the prevalence of internet addiction was estimated at 16.9%, with higher scores associated with online gaming, social media use, and non-directed internet browsing (22).

#### Older people

Although most research on problematic digital technology use focuses on children and adolescents, older adults also represent a vulnerable group with regard to the consequences of functioning in digital environments. In contrast to younger users who have grown up with digital technologies, many older adults acquire digital competencies later in life, which may shape their patterns of use and increase susceptibility to risks related to online safety, privacy, and self-regulation (23).

Problematic Internet use in older adults may also differ from patterns observed in younger populations. A comparative study of 264 Internet users aged over 60 years from Poland and Switzerland examined associations between internet use, problematic online behaviors, impulsivity, and psychological wellbeing. The findings showed that higher levels of problematic internet use were associated with lower wellbeing and with using the internet as a coping strategy for negative emotions or life dissatisfaction. The authors also noted that older adults remain an underrepresented group in research on technology-related addictive behaviors (23).

Older adults are particularly vulnerable to digital risks associated with digital exclusion (the digital divide), which results from unequal access to digital technologies and substantial differences in digital literacy and digital skills. Digital exclusion may also limit access to healthcare, public services, and social participation, thereby contributing to health inequalities among older adults (24).

Although Internet use among older adults has steadily increased, pronounced intergenerational differences persist. According to Eurostat data, in 2020, 61% of individuals aged 65–74 in the European Union used the Internet, compared with 98% of those aged 16–24 (25). A further key issue concerns digital skills levels. European data indicate that only approximately 25% of individuals aged 65–74 possess at least basic digital skills, which may limit their ability to use online services safely and increase vulnerability to online fraud, misinformation, and privacy breaches (26).

Polish older adults are among the lowest-ranked groups in the European Union in terms of digital skills (27). At the same time, digital technologies may also play a protective role by supporting social connectedness, access to health information, and the use of public and healthcare services. Among Polish Internet users aged 65+, 51% have a social media account (39% in 2023), and 80% use messaging applications (an increase of 8 percentage points compared with 2023). Although Internet use among older adults is increasing, they tend to engage with digital technologies in a more passive, consumption-oriented manner than younger users, with lower participation in creative and transactional activities (24). Therefore, preventive interventions in this group should go beyond mitigating digital risks and also focus on strengthening competencies that support safe, informed, critical, and active engagement in digital environments (27).

It appears to be significantly less dangerous in senior age, when cognitive functions decline and dopamine deficiencies make digital stimulation attractive and compensatory. However, loneliness and declining cognitive functions can become a slippery slope, among other things, replacing moderate physical activity, which is important at this age for maintaining health (28).

#### Individuals with mental health vulnerabilities

Individuals with mental health problems represent another group particularly vulnerable to the adverse consequences of problematic digital technology use. Evidence suggests a bidirectional relationship between psychological functioning and online behaviors: depressive symptoms, anxiety, impulsivity, and difficulties in emotion regulation may increase the risk of excessive Internet use, while problematic engagement with digital technologies may further exacerbate mental health difficulties. Digital technologies may also be used as a coping strategy for stress, loneliness, or negative emotions (29).

Epidemiological evidence indicates a higher prevalence of problematic internet use among individuals with mental health symptoms. A meta-analysis of 43 studies including 33,671 participants demonstrated a significant association between problematic Internet use and symptoms of depression, anxiety, and psychological distress. Individuals with elevated depressive symptoms were approximately twice as likely to exhibit problematic Internet use compared with those without such symptoms [odds ratio (OR) = 2.0; 95% CI: 1.7–2.4] (18).

Among individuals with mental disorders, difficulties in behavioral regulation and impulse control appear particularly important. Problematic internet use frequently co-occurs with attention-deficit/hyperactivity disorder (ADHD), anxiety disorders, obsessive-compulsive disorder, and mood disorders. Clinical populations may also be more susceptible to reinforcement mechanisms embedded in digital environments, such as immediate rewards, continuous stimulation, and the use of online activities as a strategy for avoiding or regulating negative emotions (29, 30).

#### Socioeconomically disadvantaged populations

Individuals in disadvantaged socio-economic circumstances represent a population particularly vulnerable to digital risks. The digital divide is commonly conceptualized at three levels, with the second and third being most relevant here. The first level refers to disparities in access to computers and the Internet; the second concerns inequalities in digital skills, competencies, usage patterns, and acceptance of digital technologies; and the third focuses on differential outcomes of digital engagement, particularly the extent to which individuals can benefit from technology use in practice (31). Evidence suggests that lower socioeconomic status, lower educational attainment, and limited material resources are significant predictors of reduced digital competencies, increasing the risk of exclusion from digital health, educational, and social services (32).

Analyses from European Union countries show persistent inequalities in digital competencies. Eurostat data indicate that in 2023 only 56% of people aged 16–74 in the EU had at least basic digital skills, with lower levels among individuals with lower educational attainment and poorer socioeconomic status (33). People with lower income also use advanced digital services, including e-health platforms, online banking, and e-government services, less frequently, which may reinforce existing social and health inequalities. In Poland reported that 60% of individuals with higher education use online public services, compared with only 2% among those with primary education (34).

Digital inequalities are particularly relevant for access to health information and preventive services. Evidence shows that individuals with lower socioeconomic status often have lower digital health literacy, understood as the ability to access, evaluate, and use online health information. Lower educational attainment is associated with greater vulnerability to health misinformation, misinterpretation of online content, and reduced ability to use digital health tools (32). At the same time, digital technologies can help reduce inequalities by improving access to health education, telehealth, and social support, provided that adequate accessibility and user skills are ensured.

#### Synthesis of key risk and protective factors

Across the populations and digital risks examined, several recurring risk and protective factors can be identified. Risk factors operate at multiple and interconnected levels, including individual characteristics, interpersonal relationships, digital environment and design, and broader social and structural conditions. Individual-level factors include younger age and ongoing neurodevelopment, impulsivity, difficulties in self-regulation and emotion regulation, pre-existing mental health difficulties, loneliness, and limited digital literacy. At the interpersonal level, limited social support, family difficulties, and insufficient parental or educational guidance may increase vulnerability. Digital environment-related factors include highly engaging platform and game design, continuous availability, reward mechanisms, notifications, social comparison, and features that may encourage prolonged or uncontrolled use. At the structural level, socioeconomic disadvantage, educational inequalities, digital exclusion, and limited access to reliable information may further increase vulnerability.

Conversely, protective factors include adequate digital literacy and health literacy, self-regulation skills, supportive family and peer relationships, appropriate parental and school-based guidance, access to mental health and social support, and the availability of safe and ethically designed digital environments. Importantly, the relative contribution of these factors differs across populations and digital risks. This supports the need for a multilevel approach in which individual competencies are complemented by family-, school-, community-, technological-, and policy-level measures.

### Selected digital risks and their implications for public health

#### Problematic Internet use and problematic social media use

Problematic Internet use and problematic social media use are among the most widely studied digital risks in public health. They are characterized by impaired control over technology use and continued online engagement despite negative psychosocial consequences. Although their classification as behavioral addictions remains debated, both have been consistently associated with poorer mental wellbeing, symptoms of depression and anxiety, and impaired social functioning (3).

These behaviors are particularly prevalent among adolescents. In Italy, 85.8% of adolescents aged 11–17 regularly use smartphones, over 72% access the Internet via smartphones, and nearly half spend more than 3 h daily on these devices, primarily using social media (35). The 2025 report *Children and Smartphones, Tablets and Computers* estimated that 5.5 million children and adolescents in Poland use Internet-connected screen devices. Around the age of four, users begin to outnumber non-users. Among adolescents aged 12–17 years, 29% use smartphones for more than 4 h daily, while 22% report 3–4 h of daily use. Screen time is also socially patterned, with longer use observed among children from lower socioeconomic backgrounds (36). A meta-analysis including nearly 42,000 adolescents found that problematic smartphone use was associated with an increased risk of depression, anxiety, perceived stress, and poor sleep quality (3). Another meta-analysis of 113 epidemiological studies involving almost 700,000 participants from 31 countries estimated the prevalence of problematic Internet use at 7.0% and Internet gaming disorder at 2.5% in the general population (2).

#### Gaming disorder and problematic gaming

Problematic gaming and gaming disorder are important digital health risks, particularly among children, adolescents, and young adults. In 2019, the WHO recognized gaming disorder in the ICD-11 as a pattern of impaired control over gaming, increasing priority given to gaming over other activities, and continued gaming despite negative consequences for personal, social, educational, or occupational functioning (37).

Epidemiological studies show that the prevalence of problematic gaming varies by age, sex, region, and diagnostic criteria. A meta-analysis of 53 studies involving more than 226,000 participants from 17 countries estimated the global prevalence of gaming disorder at 3.05%, with higher rates among males and younger individuals (38). Another meta-analysis reported a prevalence of internet gaming disorder of 4.6%, with higher rates among male adolescents than females (39).

The development of problematic gaming is influenced by game design features that promote prolonged engagement, including reward systems, social competition, continuous progression, and microtransactions. Many of these features rely on dark patterns (deceptive design techniques) that prioritize commercial goals over users' interests by encouraging engagement through manipulation or excessive persuasion rather than informed choice. Such practices may undermine user autonomy and increase the likelihood of prolonged or unintended use of digital services, highlighting the importance of ethical design in digital health promotion and prevention (40). Gaming disorder is consistently associated with depression, anxiety, sleep disturbances, and impaired psychosocial functioning (38). Individual factors, including impulsivity, poor emotion regulation, and using gaming to cope with stress, further increase the risk.

#### Cyberbullying and online sexual risks

Cyberbullying and online sexual risks are major digital harms affecting children and adolescents. Cyberbullying involves repeated harmful behaviors through digital technologies, including harassment, dissemination of offensive content, online social exclusion, and non-consensual sharing of personal materials. Compared with offline bullying, it may have a wider reach, greater persistence, and involve anonymous perpetrators (41).

Cyberbullying is common among young Internet users. A meta-analysis of 63 studies involving more than 330,000 adolescents reported cybervictimization rates ranging from 14% to 57.5%, with verbal aggression being the most frequent form (42). Victims are at increased risk of depression, anxiety, low self-esteem, and suicidal ideation (38). Online sexual risks include grooming, unwanted sexual solicitations, coercive sexting, and sextortion. A meta-analysis of adolescents aged 12–17 years found that about 15% had received unwanted sexual solicitations online and 12% had been exposed to unwanted sexual content. Younger adolescents, those with limited social support, and those with lower digital safety skills appear particularly vulnerable (43).

#### Online gambling and financial risks

Online gambling is an important digital health risk due to the widespread availability of gambling platforms, anonymous participation, and engagement-enhancing design features. Continuous access to online casinos, sports betting, and other gambling activities via mobile devices may increase gambling frequency and intensity. Problematic online gambling is recognized as a behavioral addiction characterized by impaired control, persistent gambling despite negative consequences, and substantial psychological, social, and financial harms (44).

Easy access, rapid financial transactions, and 24-h availability further increase the risk of problematic gambling (45). A systematic review of 44 studies found that the prevalence of problem gambling among adolescents ranged from 0.2% to 12.3%, with estimates of 2.1–2.6% in the United States and 0.2–12.3% in Europe (46).

An emerging concern is the convergence of online gambling and video gaming. Features such as loot boxes (virtual reward boxes) share structural similarities with gambling and may encourage financially risky behaviors, particularly among young people. More frequent loot box use has been associated with greater gambling severity, although the causal relationship remains unclear (47).

#### Cybersecurity threats and cybercrime

Digital security threats and cybercrime are major digital risks associated with the widespread use of digital technologies. These include phishing, identity theft, data breaches, financial fraud, and privacy violations, resulting in financial, psychological, and social harms (48).

The increasing digitalization of financial, administrative, and healthcare services has expanded exposure to cyber threats. According to the European Union Agency for Cybersecurity (ENISA), phishing remains one of the most common forms of cyberattack (49). Older adults and individuals with lower digital literacy are particularly vulnerable because they may have greater difficulty identifying fraudulent communications, protecting personal data, and assessing the credibility of online information.

### Building digital hygiene promotion strategies: implications for prevention and public health practice

The growing prevalence of digital risks underscores the need for preventive strategies that go beyond reducing screen time. Digital hygiene should promote safe, healthy, and informed use of digital technologies by strengthening individual competencies and implementing supportive environmental and system-level interventions. As in other areas of health promotion, effective prevention requires a multilevel approach addressing psychological, social, educational, and technological determinants (50).

#### Developing digital literacy and self-regulation skills

Strengthening digital literacy and self-regulation is a core component of digital hygiene. Digital literacy includes not only technical skills but also the ability to critically evaluate online information, protect privacy, recognize digital threats, and make informed decisions in digital environments (51). Higher digital literacy is associated with lower risks of problematic Internet use and greater adoption of online safety behaviors. Self-regulation—the ability to manage screen time, regulate emotions, and control impulsive online behaviors—is another key protective factor, with deficits consistently linked to problematic internet use, social media use, and gaming (52).

From a public health perspective, digital hygiene interventions should strengthen both digital literacy and self-regulation across schools, families, and communities. These programs should be age-appropriate and combine technical education with psychosocial skills that promote safe, healthy, and balanced use of digital technologies (53).

#### Family and educational interventions

Families and schools play a central role in promoting safe digital practices and developing the competencies needed to navigate online environments. Evidence indicates that active parental mediation—including open communication, jointly established rules for technology use, and modeling responsible digital behaviors—is associated with a lower risk of problematic Internet and social media use among children and adolescents (54). Compared with restrictive approaches, supportive strategies based on education, trust, and the development of autonomous decision-making skills appear to be more effective.

School-based programs that promote digital literacy, online safety, and social-emotional skills improve online behaviors and awareness of digital risks. Such interventions reduce cyberbullying perpetration by approximately 9–15% and victimization by 14–15% (55). The greatest benefits are observed in multicomponent programs involving students, teachers, and parents and integrating digital education with broader health promotion initiatives.

#### Public health approaches and community-based prevention

Effective prevention of digital risks requires a population-based public health approach that goes beyond individual-level interventions. Prevention should integrate health promotion, education, surveillance, and collaboration across healthcare, education, public administration, civil society, and the technology sector. Such a comprehensive approach can reduce digital harms while strengthening protective factors, including digital literacy, psychological resilience, and healthy technology use (11, 56).

A key component of public health is the implementation of preventive programs for populations at increased risk, including children and adolescents, older adults, and individuals with limited digital literacy. Community-based interventions involving schools, families, healthcare providers, and non-governmental organizations are more effective than those targeting individual behavior alone (57). Continuous surveillance of digital risks and evaluation of preventive programs are also essential to ensure that interventions remain effective and responsive to changing needs (58).

Digital hygiene should be integrated into public health and education policies to promote safe digital environments and reduce inequalities in digital literacy and access to prevention. This approach is consistent with the socio-ecological model, which emphasizes interactions between individuals and their environments at the family, school, community, and policy levels (59).

#### Creating safer digital environments

Responsibility for reducing digital risks should be shared by users, public institutions, digital service providers, and technology designers. Public health approaches increasingly emphasize safety by design and privacy by design to improve user safety, protect personal data, and promote wellbeing (9, 11).

Key strategies include limiting exposure to harmful content, strengthening data protection, increasing transparency of content recommendation algorithms, and providing tools such as screen time monitoring, privacy settings, parental controls, and abuse-reporting mechanisms (60). Their effectiveness is greatest when combined with educational interventions that strengthen digital competencies. A meta-analysis of 11 studies found that interventions improved parents' digital safety knowledge and skills and were associated with reduced screen time among both children and parents (61).

#### Digital safety among older adults

Older adults are one of the key target groups for digital safety interventions. As public services, healthcare, and banking become increasingly digital, older people rely more on online technologies while remaining particularly vulnerable to phishing, identity theft, financial fraud, and misinformation. This vulnerability is largely driven by lower digital literacy, limited experience with online services, and difficulties in assessing information credibility (9, 11).

Digital hygiene strategies should therefore include education on recognizing cyber threats, using e-banking and e-health services safely, protecting personal data, and evaluating online information (62). Strengthening older adults' confidence and self-efficacy in using digital technologies is also important, as it promotes digital inclusion, supports independent living, and reduces the risk of cyber fraud (63).

### Implications for public health policy

The presented overview of digital threats suggests that effective digital hygiene prevention cannot rely on a uniform, one-size-fits-all model, but must account for the heterogeneous distribution of risk factors and resources across population groups. Children and adolescents, older adults, and individuals in disadvantaged socioeconomic positions differ both in the types of digital risks they face and in the mechanisms underlying their vulnerability. These range from the developmental immaturity of self-regulatory neurobiological systems in younger users to lower digital literacy and increased susceptibility to fraud among older adults (23, 27), and structural barriers to access and skills associated with second- and third-level digital divide (31). This implies that prevention strategies focused solely on reducing screen time or relying on generic educational campaigns do not adequately address the needs of these populations. Instead, they should be replaced by differentiated, yet coherently framed, public health approaches to digital hygiene promotion, as outlined in Table 1.

**Table 1:** Selected digital risks by most affected populations and levels of preventive intervention.

Digital risk	Most affected/vulnerable populations	Individual level (digital hygiene)	Family & school level (meso)	Community & policy level (macro)
Problematic internet & social media use	Adolescents; people with depression, anxiety, ADHD, emotion-regulation difficulties; older adults using the internet to cope	Digital literacy, self-regulation, screen-time management, critical evaluation of content	Active parental mediation; jointly set rules; school programmes building psychosocial skills	Safe-by-design platforms; algorithm transparency; integration into public health strategies
Gaming disorder & problematic gaming	Children, adolescents, young adults; disproportionately males	Awareness of engagement mechanics; impulse and emotion regulation; balanced use	Parental modeling and dialogue; school-based prevention integrated with health promotion	Regulation of dark patterns, loot boxes, microtransactions; design accountability of providers
Cyberbullying & online sexual risks	Children and adolescents; those with low social support and low digital-safety competencies	Recognizing harm; help-seeking; protective online behaviors	Multicomponent school programmes (students, teachers, parents); social-emotional learning	Reporting mechanisms; content moderation; child-protection frameworks
Online gambling & financial risks	Adolescents and young users (esp. via loot-box/gaming convergence)	Awareness of gambling-like mechanics; financial-risk literacy	Parental awareness of in-game spending; school financial/digital education	Age verification; advertising limits; regulation of gambling–gaming convergence
Cybersecurity threats & cybercrime	Older adults; people with low digital literacy and low socioeconomic status	Recognizing phishing/fraud; safe use of e-banking/e-health; data protection	Peer/family support in verifying information and services	Targeted programmes for older adults; institutional shift of responsibility for digital safety; ongoing surveillance

Low socioeconomic status and digital exclusion act as cross-cutting amplifiers of vulnerability across all risk categories.

The analysis indicates that public health policy addressing digital risks should be based on a multilevel model that captures bidirectional interactions between individuals and their environments at both the micro level (family, peers, school) and the macro level (local communities, societal attitudes, and legal regulations). This requires sustained cross-sector collaboration involving healthcare systems, education, public administration, non-governmental organizations, and the technology sector, as well as a systematic shift in responsibility for digital safety from individual users to institutions and technology providers (11, 31).

Community-based interventions have been shown to be more effective than those targeting individuals alone therefore, digital hygiene promotion should be formally integrated into national and local public health strategies, alongside other areas of behavioral addiction prevention. A key condition for effectiveness is the simultaneous addressing of structural inequalities in access to technology, digital competencies, and the ability to benefit from digital resources, as interventions focused solely on individual education may inadvertently reinforce rather than reduce disparities. Continuous surveillance of digital risks and systematic evaluation of preventive programs are also essential to ensure that public health policy can be adapted to the rapidly evolving digital environment and the diverse needs of population groups (57).

According to the theory of ecological systems, when thinking about humans, we should treat them as part of a complex network of connections, and understanding should occur at different levels in order to increase the effectiveness and adequacy of the solutions undertaken. This is precisely how we propose thinking about ways to deal with risk factors. Decision-makers should use protective tools at every level of human activity and shape regulations in a way that affects society in as many aspects as possible. Research shows that different groups of citizens require different types of influence, so to achieve the desired effect and reduce the negative consequences of excessive use of new technologies, various forms of intervention should be applied. This task does not seem easy, but its significance is enormous and will likely influence how the next generation experiences life, thinks, and acts, so it is worth examining.

## Conclusions

The rapid development of digital technologies has made online-related risks a major public health challenge. Evidence indicates that effective prevention requires digital hygiene promotion integrating digital literacy, self-regulation, education, and safe digital environments through cross-sector collaboration between health, education, public administration, and technology stakeholders. Digital hygiene promotion should be an integral part of public health strategies, supporting safe and responsible technology use. Further research is needed to evaluate preventive interventions and develop models tailored to diverse population needs.

## Limitations

This narrative review has several limitations. It is not a systematic review or meta-analysis, which may affect the comprehensiveness of the included evidence. The analysis covers selected public health–relevant digital risks, while the broader spectrum of digital transformation-related risks is wider and rapidly evolving. Differences in definitions, measurement tools, and study populations also limit comparability across studies. Finally, digital hygiene promotion remains an emerging concept requiring further research on effective preventive models.
